# Grassland structural heterogeneity in a savanna is driven more by productivity differences than by consumption differences between lawn and bunch grasses

**DOI:** 10.1007/s00442-016-3698-y

**Published:** 2016-08-13

**Authors:** Michiel P. Veldhuis, Heleen F. Fakkert, Matty P. Berg, Han Olff

**Affiliations:** 1 Groningen Institute for Evolutionary Life Sciences, University of Groningen, P.O. Box 11103, 9700 CC Groningen, The Netherlands; 2 Department of Ecological Science, VU University Amsterdam, De Boelelaan 1085, 1081 HV Amsterdam, The Netherlands

**Keywords:** Nutritional quality, Grassland mosaic, Primary production, Grazing, Hluhluwe-iMfolozi Park

## Abstract

Savanna grasslands are characterized by high spatial heterogeneity in vegetation structure, aboveground biomass and nutritional quality, with high quality short-grass grazing lawns forming mosaics with patches of tall bunch grasses of lower quality. This heterogeneity can arise because of local differences in consumption, because of differences in productivity, or because both processes enforce each other (more production and consumption). However, the relative importance of both processes in maintaining mosaics of lawn and bunch grassland types has not been measured. Also their interplay been not been assessed across landscape gradients. In a South African savanna, we, therefore, measured the seasonal changes in primary production, nutritional quality and herbivore consumption (amount and percentage) of grazing lawns and adjacent bunch grass patches across a rainfall gradient. We found both higher amounts of primary production and, to a smaller extent, consumption for bunch grass patches. In addition, for bunch grasses primary production increased towards higher rainfall while foliar nitrogen concentrations decreased. Foliar nitrogen concentrations of lawn grasses decreased much less with increasing rainfall. Consequently, large herbivores targeted the biomass produced on grazing lawns with on average 75 % of the produced biomass consumed. We conclude that heterogeneity in vegetation structure in this savanna ecosystem is better explained by small-scale differences in productivity between lawn and bunch grass vegetation types than by local differences in consumption rates. Nevertheless, the high nutritional quality of grazing lawns is highly attractive and, therefore, important for the maintenance of the heterogeneity in species composition (i.e. grazing lawn maintenance).

**Electronic supplementary material:**

The online version of this article (doi:10.1007/s00442-016-3698-y) contains supplementary material, which is available to authorized users.

## Introduction

Savanna grasslands are characterized by high spatial heterogeneity, with a diverse species assemblage that exhibits a wide variety of plant traits. Based on these traits, two functionally distinct communities can be identified. Grazing lawn patches, existing of short (0–20 cm) stoloniferous grass species with high foliar nutrient concentrations (McNaughton 1984; Stock et al. 2010; Hempson et al. 2014) and bunch grassland patches, consisting of medium/tall (>30 cm) and generally nutrient-poor grass species. This differentiation results in lawn-bunch mosaics that exhibit high spatial heterogeneity in both food quantity and quality for herbivores and have important implications for other trophic levels. These mosaics can promote resource partitioning among savanna herbivores (Voeten and Prins 1999; Farnsworth et al. 2002; Olff et al. 2002; Cromsigt and Olff 2006; Kleynhans et al. 2011; Kartzinel et al. 2015), buffer herbivore populations dynamics against temporal variation in resources (Walker et al. 1987; Owen-Smith 2004; Hopcraft et al. 2010) and affect grasshopper (Van der Plas et al. 2012) and bird community composition (Hovick et al. 2014). Therefore, good understanding of the determinants of this type of spatial heterogeneity in vegetation structure is needed.

Previous research has given strong attention to explaining differences in nutritional quality between lawn and bunch grasses, emphasizing the key role for large grazing herbivores. Defoliation by grazers has been shown to increase foliar nutrient concentrations of lawn grasses through promoting fresh regrowth, keeping plants in a physiologically young active stage (McNaughton 1976; Hik and Jefferies 1990; McNaughton et al. 1997a; Ruess et al. 1997). Also, local deposition of dung and urine acts as a natural fertilizer (Detling and Painter 1983; Ruess and McNaughton 1984; Frank and McNaughton 1993; McNaughton et al. 1997b; Frank and Groffman 1998; Augustine et al. 2003). Furthermore, high litter quality, as a result of dominance of high nutritional quality grass species, results in high soil nutrient turn-over through fast decomposition rates (Wedin and Tilman 1990; Grime et al. 1996; Wedin and Tilman 1996; Olofsson and Oksanen 2002; Coetsee et al. 2011; Sjogersten et al. 2012). Finally, decreased soil moisture availability resulting from defoliation and soil compaction, through increased evaporation and decreased infiltration rates, by large herbivores can result in increased foliar nutrient concentrations (Veldhuis et al. 2014). As large herbivores generally prefer higher quality forage, such nutritional quality differences that arise through either of these mechanisms are expected to lead to differences in consumption rates by herbivores, promoting vegetation structural heterogeneity.

In contrast, much less data are available on the importance of productivity differences between lawn and bunch grass-dominated patches in causing vegetation structural heterogeneity. Grazing lawn primary productivity remains at remarkably high levels under such high grazing intensities (Bonnet et al. 2010), sometimes even higher than less intensively grazed bunch grass patches under (spatially separated) similar rainfall conditions (McNaughton 1985), probably as a result of compensatory growth or enhanced nutrient availability. In contrast, Veldhuis et al. (2014) suggest that herbivore-induced drought in grazing lawns can reduce their productivity in comparison with adjacent bunch grasslands.

It is evident that the spatial differences in amount of standing biomass (and hence heterogeneity) will be determined by a combination of spatial differences in primary production and herbivore consumption. However, the relative contribution of these two processes in the formation of grazing mosaics remains unknown. So far, primary production and herbivore consumption of lawn and bunch grasses have been studied in isolation or in spatially separated areas (McNaughton 1985; Person et al. 1998; Bonnet et al. 2010) which makes it impossible to determine whether differences found are due to characteristics of both vegetation types or differences in environmental conditions (soil nutrients, water availability). This can only be done when rates of productivity and consumption of grazing lawns and nearby adjacent bunch grass patches are compared in the same ecosystem.

When planning such a comparison, it is important to note the original definition of grazing lawns as a distinct plant community with intrinsic trait differences related to dwarfing: e.g. short statured and often stoloniferous/rhizomatous species (McNaughton 1984). Heavily grazed areas/patches of inherently tall species (different structure) and grazing lawns (both different structure and different species composition) are often mixed up in the literature causing confusion on underlying mechanisms. For our study, we adopt the original definition of grazing lawns, which are characterized by both a different vegetation structure and different species composition, of the stoloniferous growth form.

Grassland productivity in tropical savannas is generally positively related to short term (Bonnet et al. 2010) and long term rainfall (McNaughton 1985; Fritz and Duncan 1994; O’Connor et al. 2001). Rainfall is highly variable in savanna ecosystems in both space and time (McNaughton 1985; Bonnet et al. 2010). Furthermore, plant developmental stages (vegetative growth, flowering, nutrient resorption) are expected to affect plant nutritional quality. For example, post-burn green flush of bunch grasses in the early wet season is known to attract large numbers of herbivores to these palatable highly productive areas (Wilsey 1996; Gureja and Owen-Smith 2002), while later in the wet season herbivores make profitable use of grazing lawns (Kleynhans et al. 2011; Yoganand and Owen-Smith 2014). Therefore, the relative importance of production and consumption differences between lawn and bunch grasses may vary along landscape rainfall gradients, and with the progression of the growing season.

In this study, we, therefore, quantified along a landscape rainfall gradient the differences between nearby lawn and bunch grass patches in (i) primary productivity (ii) nutritional quality, (iii) herbivore consumption (iv) the percentage of the productivity consumed by herbivores. This allowed the assessment of the relative importance of different mechanisms that cause vegetation structural heterogeneity in this savanna ecosystem.

## Materials and methods

We conducted our study in the the Hluhluwe–iMfolozi Park (HiP), (28°00′–28°26′S, 31°43′–32°00′E) an 897-km^2^ reserve in KwaZulu-Natal, South Africa from September 2013 till July 2014. Mean annual rainfall ranges from ca. 500 mm (iMfolozi) to over 900 mm (Hluhluwe), with a wet season spanning from October till March. Vegetation consists mostly of mixed patches of forest, grassland, thicket and savanna. Dominant large herbivores include white rhino (*Ceratotherium simum*), buffalo (*Syncerus caffer*), zebra (*Equus burchelli*), wildebeest (*Connochaetes taurinus*), warthog (*Phacochoerus africanus*) and impala (*Aepyceros melampus*) (Ezemvelo KZN Wildlife census data 2014, unpublished).

### Site selection and preparation

**Fig. 1:**
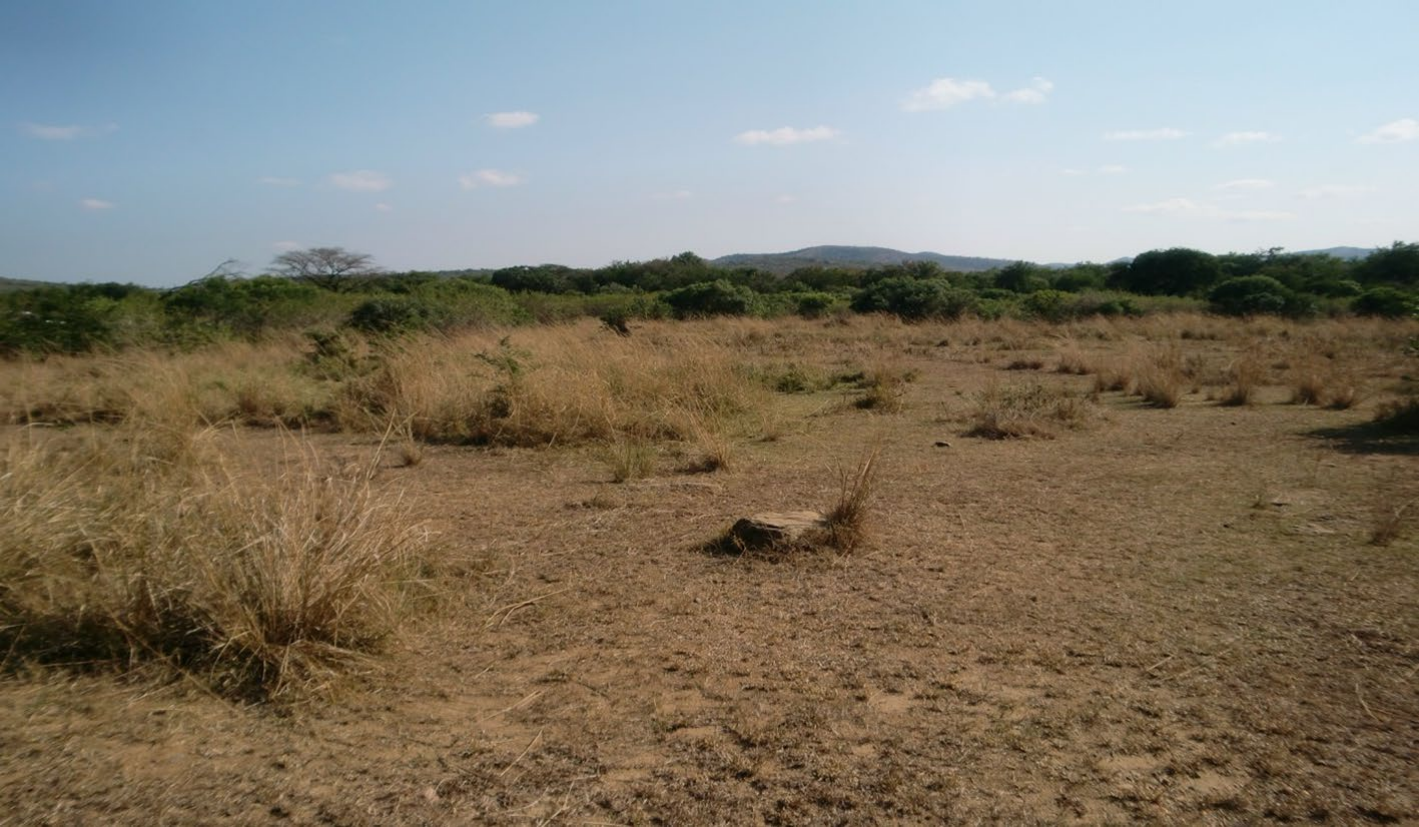
Structural heterogeneity in the grass layer of an African savanna ecosystem in Hluhluwe-iMfolozi Park, South Africa. Color version available online. Photo credit: Michiel Veldhuis

Seven sites were chosen based on rainfall maps to obtain large differences in annual rainfall between sites (Online resource 1). Sites consisted of continuous layers of bunch grasses interspersed grazing lawns (Fig. 1). Lawn grass cover varied between 17 and 40 % with the exception of the two highest rainfall sites where lawn grass patches were absent. Woody cover varied between 12 and 40 % cover.

Fire is a common disturbance in African savannas, which affects primary production and consumption by herbivores. We chose to burn all the sites for two reasons. First, we wanted to create similar starting conditions for lawn and bunch grasslands. Grazing lawns typically have almost no above-ground biomass at the end of the dry season. Similar starting conditions for bunch grasses could be obtained by either clipping or burning, where we chose for the latter one for practical reasons since it has been demonstrated that burned and clipped treatments do not significantly differ in primary production (Van de Vijver et al. 1999). Second, the mean (3.8 years) and median (1.8 years) fire return periods for the study area represent relative high fire frequencies and on average over 25 % of the park is burned annually (Balfour and Howison 2002). Large herbivores, therefore, can practically always choose to forage in burned areas, which is likely the case due to the “magnet effect” of the green flush (Archibald et al. 2005). To compare consumption rates between lawn and bunch grasses we, therefore, judged it would be more appropriate to burn the sites at the onset of the experiment. Most sites (*n* = 5) and their surrounding were burned as part of the park management plan. The remaining two sites (the lowest and highest in rainfall) were burned down resulting in ca. 75 × 75 m burned area surrounded by unburned vegetation.

### Rainfall

Rain gauges were installed at every site and emptied once every 2 weeks. A few ml of sunflower oil was poured into the rain gauge to prevent evaporation. We used rain gauge data from nearby sites to fill gaps in rainfall data in case rain gauges were destroyed by animals and subsequently installed new rain gauges. Rainfall data were summed in periods to synchronize them with measurements on primary production and consumption.

### Primary production and consumption

Primary production and consumption of both lawn and bunch grasses were quantified using movable cages (McNaughton et al. 1996). On each site, we established three iron cages of 1 × 1 × 1 m on both lawn and bunch grass areas. These areas were identified based on species composition and associated difference in vegetation structure, where lawn grass areas were dominated by *Digitaria longiflora, Sporobolus nitens, Panicum coloratum, Urochloa mosambicensis, Dactyloctenium australe* and *Cynodon dactylon*. Bunch grass areas were dominated by *Sporobolus pyramidalis, Themeda triandra, Eragrostis curvula, Panicum maximum, Digitaria eriantha, Setaria sphacelata, Cymbopogon excavatus, Hyparrhenia filipendula, Chloris gayana* and *Bothriochloa insculpta*.

Each iron cage was wrapped in chicken wire netting (2.5 cm mesh) to prevent access to all herbivores larger than mice, and fixed to the ground using tent pegs on the bottom to prevent toppling. At the start of the experiment aboveground biomass in an area of 40 × 40 cm just next to the cage was clipped to determine initial biomass (initial). Subsequently, at the end of each sample period both inside (caged) and outside (grazed) the cage another 40 × 40 cm area was clipped after which the cage was moved to a comparable area within the same vegetation type. For subsequent sample periods biomass clipped in the grazed treatment was used as the initial biomass estimate for the next period. Periods between moving the cages differed from 20 to 42 days between September 2013 and May 2014, with shorter periods during the wet season where production and consumption were expected to be highest. A final measurement was taken halfway July 2014 in the middle of the dry season. All clipped biomass samples were labeled and taken back to the laboratory where they were dried (48 h at 70 °C), weighed, and ground (Foss Cyclotec, 2 mm) to determine chemical composition.

### Chemical composition

Carbon (%C) and nitrogen (%N) content of aboveground biomass were estimated using a Bruker near-infrared spectrophotometer (NIR, Ettlingen) using a multivariate calibration (frequency range 11,602–3602 cm^−1^ for both C and N) of foliar samples measured both on the NIR and CHNS EA1110 elemental analyzer (Carlo-Erba Instruments, Milan). Cross-validation showed these NIR predicted C and N content are highly accurate (*R*^2^ = 95.7 for N, *R*^2^ = 92.9 for C, *N* = 1759).

## Data analysis

### Data preparation

Aboveground net primary productivity (ANPP) was calculated as the difference in dry weight biomass inside the cage at the end of a sample period and the initial biomass outside the cage at the start of each period. Herbivore consumption was calculated as the difference in dry weight biomass inside and outside the cage at the end of each period. We averaged primary productivity and consumption at each site for each time period to deal with spatial pseudo-replication and to overcome problems in calculating annual and cumulative productivity and consumption due to missing data (9 out of 288 cage periods) as a result of cage toppling. Annual productivity and consumption were calculated for the periods between September and May, since we found mostly negative production rates for the last period (May–July) (Online resource 2). We, therefore, judged measurements from this latter period as unreliable, likely as a result of grasses dying off during the dry season.

All statistical analyses described below started with full models and used backwards stepwise removal of non-significant terms to obtain final models. Quadratic terms were added for the explanatory variables rainfall and production, since we expected the effect sizes to decrease towards specific thresholds. In all models, assumptions of equal variances between vegetation types were violated and we modeled equal variances following Zuur et al. (2009) using the “varIdent” function within the “nlme” package (Pinheiro et al. 2014). Statistical analyses used to test for differences between vegetation types, only the 5 sites where both vegetation types were present were used. We also constructed separate models for lawn and bunch grasslands when vegetation types showed significant interactions to obtain additional insight in observed patterns. Furthermore, conditional and marginal *R*^2^ were calculated following Johnson (2014). All statistical analyses were executed in R 3.1.2 (R Core Team 2015).

### Primary productivity

We studied the effect of rainfall and vegetation type on primary productivity in three ways: annual primary production (from September to May), periodic primary production (using every period as separate data points) and cumulative primary production (from September to the end of every period). Annual primary production was modeled using analysis of covariance (ANCOVA) with vegetation type and annual rainfall as explanatory variables. Subsequently, we constructed linear mixed effect models (LMM’s) for periodic primary production and cumulative primary production with corresponding rainfall periods and vegetation type as fixed effects. Time was used a random effect nested within Site to deal with the temporal pseudo-replication (repeated measured over time resulting in non-independent errors).

### Nutritional quality

Logarithmic transformations of foliar N content and C:N ratios were highly correlated (*R*^2^ = 0.99). We, therefore, decided to use foliar N content as a measure of nutritional quality for further analyses and used log-transformation to meet assumptions of normality. LMM’s were used to investigate effects on nutritional quality throughout the season. Fixed effects were vegetation type, periodic and cumulative rainfall and all interactions. Time was used as a random effect with Cage ID nested within Site.

### Herbivore consumption

Herbivore consumption was analyzed in similar way as primary production with three response variables (annual consumption, periodic consumption and cumulative consumption). ANCOVA was used to investigate the effect of vegetation type and annual production on annual herbivore consumption. Subsequently, LMM’s were constructed to test the dependence of periodic consumption and cumulative consumption, with Time as random effect nested within Site. For periodic consumption we used vegetation type, periodic production, foliar N content and all interactions as fixed effects. Full model for cumulative production comprised both vegetation type and cumulative production as fixed effects.

### Percentage production consumed

We calculated the percentage of the primary production that was consumed by large herbivores both on an annual basis and throughout the season using the cumulative production and consumption estimates. ANCOVA was used to investigate the effect of vegetation type and annual production on the percentage consumed by large herbivores. LMM was used to test the dependence of cumulative percentage consumed (the percentage of the primary production consumed by large herbivores until that point in time) on vegetation type and the cumulative production. Time was included as a random effect nested within Site.

## Results

### Primary production

**Fig. 2:**
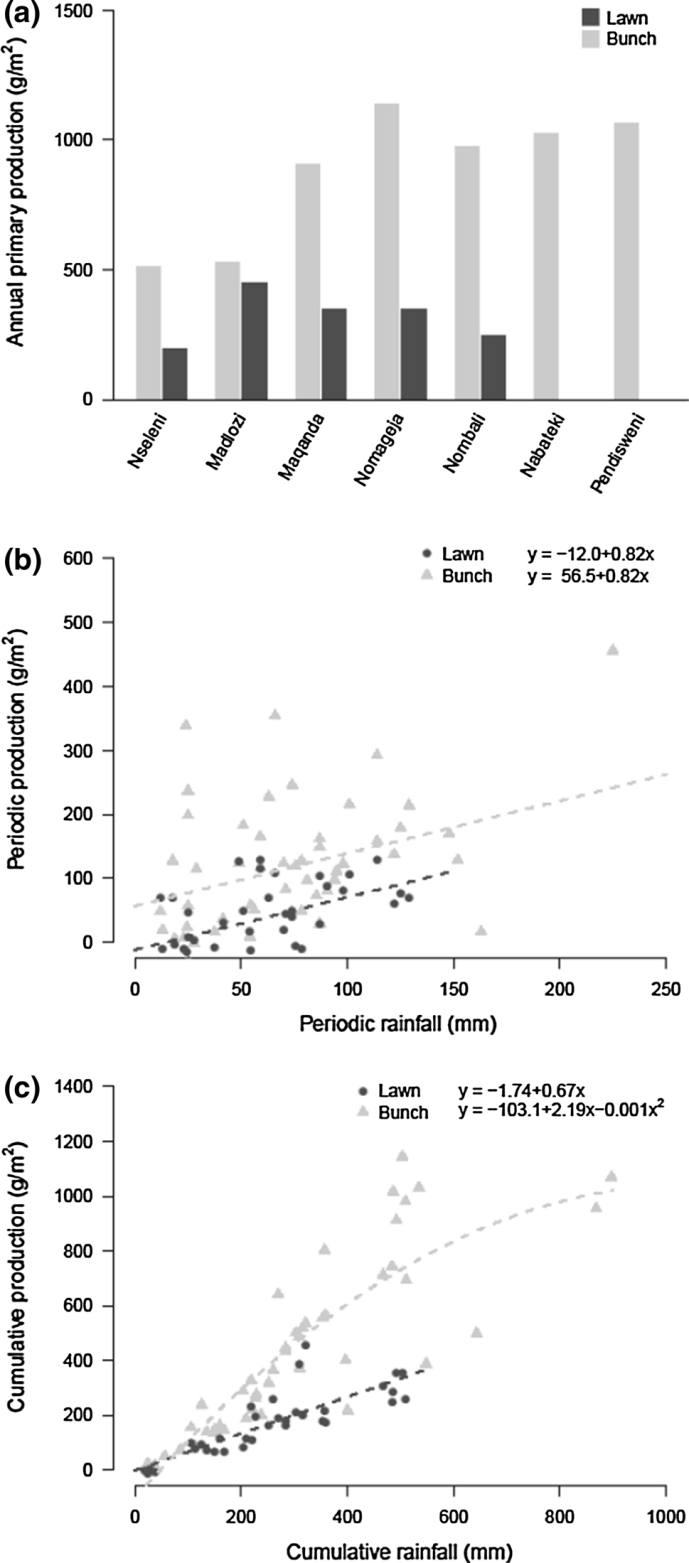
Above-ground primary production for lawn (*black*) and bunch grasses (*grey*) over a full growing season from September 2013 till May 2014. Primary production was measured using movable cages that were moved every 4–6 weeks. **a** Total primary productivity over the growing season for each of the seven sites. Sites are ordered by rainfall (see Online resources 1 and 2 for actual amounts of annual rainfall). **b** Periodic production as a function of periodic rainfall. **c** Cumulative production against cumulative rainfall

Overall, periodic primary productivity of both lawn and bunch grasses was strongly positively related to periodic rainfall (Table 1; Fig. 2b). Lawn grasses produced 0.82 g m^−2^ mm^−1^ rainfall. Bunch grasses showed similar increases in productivity with periodic rainfall (no significant interaction), but was 68.5 g m^−2^ more productive than lawn grasses, irrespective of rainfall. However, we did find a significant interaction term between vegetation type and rainfall for annual production (Table 1). Closer investigation on separate models per vegetation type (Table 2) shows that annual production in bunch grasses was positively related to annual rainfall, but leveled off with increasing amounts of rainfall towards a threshold of ca. 1000 g m^−2^ (Fig. 2a, c; Table 2, significant negative quadratic term). Annual aboveground production of lawn grasses was not related to annual amount of rainfall (Fig. 2a; Table 2). Furthermore, a significant interaction between cumulative rainfall and vegetation type indicates that bunch grasses show higher productivity under similar rainfall conditions and this difference increases with rainfall (Fig. 2c; Table 1).

**Table 1:** Overall model results for the effect of vegetation type, amount of rainfall on primary productivity and foliar [N]

Response variable	Explanatory variables	Adj. *R* ^2^	Con. *R* ^2^	Mar. *R* ^2^	*df*	Estimate	*F*	*P*
Annual production		0.90			3.6		28.1	<0.001
	Intercept					−321.3		
	Vegetation type					661.4	57.5	<0.001
	Annual rainfall					2.65	13.0	0.01
	Annual rainfall^2^							NS
	Veg. type × ann. rainfall					−2.69	13.8	<0.01
Periodic production			0.29	0.26				
	Intercept					56.5		
	Vegetation type				1.4	−68.5	16.5	0.01
	Periodic rainfall				1.59	0.82	19.7	<0.001
	Periodic rainfall^2^							NS
	Veg. type × per. rainfall							NS
Cumulative production								
	Intercept					−74.1		
	Vegetation type				1.4	63.4	17.0	0.01
	Cumulative rainfall				1.57	1.68	137.8	<0.001
	Cumulative rainfall^2^				1.57	0.0003	4.1	<0.05
	Veg. type × cum. rainfall				1.57	−1.17	35.2	<0.001
Log [N]			0.69	0.61				
	Intercept					0.633		
	Vegetation type				1.4	0.058	12.8	0.02
	Periodic rainfall				1.65	0.001	6.7	0.01
	Cumulative rainfall				1.65	−0.001	116.4	<0.001
	Veg. type × per. rainfall							NS
	Veg. type × cum. rainfall				1.65	0.0006	6.5	0.01
	Per. rainfall × cum. rainfall							NS
Annual consumption		0.83			3.6		16.0	<0.01
	Intercept					439.3		
	Vegetation type					−505.3	28.1	<0.01
	Annual production					−0.05	0.6	0.43
	Veg. type × ann. production					1.02	19.3	<0.01
Periodic consumption			0.56	0.47				
	Intercept					7.58		
	Vegetation type				1.4	6.32	7.1	0.056
	Periodic production				1.56	0.61	29.8	<0.001
	Log [N]				1.56	−9.09	11.7	<0.01
	Veg. type × per. production							NS
	Veg. type × Log [N}							NS
	Per. production × Log [N]				1.56	−0.58	7.0	0.01
Cumulative consumption			0.81	0.90				
	Intercept					−10.8		
	Vegetation type							NS
	Cumulative production				1.59	0.44	70.7	<0.001
	Cumulative production^2^							NS
	Veg. type × cum. production							NS
Annual % consumed		0.60			2.7		9.1	<0.01
	Intercept					90.9		
	Vegetation type					23.5	10.2	<0.05
	Annual production					−0.09	8.0	<0.05
	Veg. type × ann. production							NS
Cumulative % consumed			0.28	0.62				
	Intercept					27.4		
	Vegetation type				1.4	−5.62	1.8	0.24
	Cumulative production				1.51	0.05	4.3	<0.05
	Cumulative production^2^				1.51	−0.00	6.2	<0.05
	Veg. type × cum. production				1.51	0.09	5.2	<0.05

Furthermore, model results on the effect of vegetation type, primary production and foliar [N] on herbivore consumption and the percentage of primary production that is consumed. Adjusted *R* ^2^ (Adj. *R* ^2^) are given for ANCOVA models, whereas Conditional (Con. *R* ^2^) and Marginal *R* ^2^ (Mar. *R* ^2^) represent the explained variation for linear mixed effect models and corresponding degrees of freedom (*df*), estimated coefficient (estimate), *F* value (*F*) and *P* value (*P*)

**Table 2:** Overall model results separated for lawn and bunch for all models with significant interactions with vegetation type

Response variable	Lawn								Bunch						
	Explanatory variable	Adj. *R* ^2^	Con. *R* ^2^	Mar. *R* ^2^	*df*	Est.	*F*	*P*	Adj. *R* ^2^	Con. *R* ^2^	Mar. *R* ^2^	*df*	Est.	*F*	*P*
Annual production									0.89			2.4		27.2	<0.01
	Intercept					323.2							−969.8		
	Annual rainfall							NS					6.03	29.5	<0.01
	Annual rainfall^2^							NS					−0.004	24.9	<0.01
Log [N]			0.57	0.54	1.33					0.69	0.53	1.46			
	Intercept					0.77							0.65		
	Periodic rainfall							NS							NS
	Cumulative rainfall					−0.001	35.7	<0.001					−0.001	20.6	<0.001
	Per. rain × cum. rain							NS							NS
Annual consumption		0.80			1.3										
	Intercept					−66.0							344.0		
	Annual production					0.97	16.6	0.02							NS

Adjusted (Adj.) *R* ^2^ are given for ANCOVA models, whereas Conditional (Con.) and Marginal (Mar.) *R* ^2^ represent the explained variation for linear mixed effect models and corresponding degrees of freedom (*df*), estimated coefficient (estimate), *F* value (*F*) and *P* value (*P*)

### Nutritional quality

**Fig. 3:**
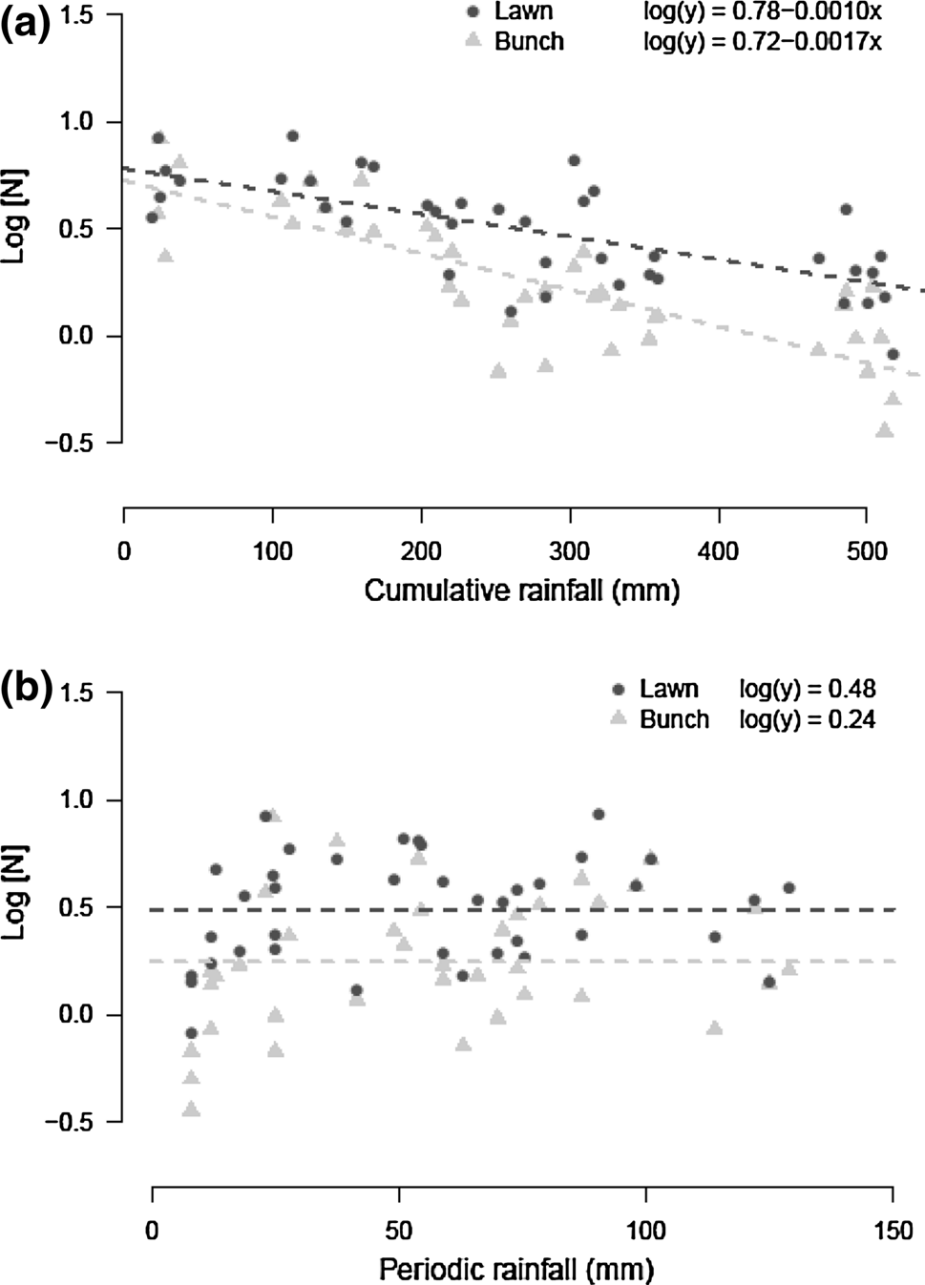
Effect of **a** cumulative rainfall and **b** periodic rainfall on foliar N concentrations, representing short and long term effects of rainfall on plant nutritional quality for lawn (*black*) and bunch grasses (*grey*)

Foliar N contents were higher for lawn than bunch grasses at any rainfall (Table 1; Fig. 3). Periodic rainfall showed mixed effect, with a positive effect on N content in the overall model (Table 1), but no effect when the analysis was split up between vegetation types (Fig. 3b; Table 2). Cumulative rainfall decreased foliar N content and this was also consistent in models for lawn and bunch grasses separately (Fig. 3a; Table 2). Furthermore, the negative effect of cumulative rainfall on N content was much larger than the positive effect of periodic rainfall (Tables 1, 2). The difference in foliar N content between the vegetation types was small at the onset of the season (0.18 % at 0 mm), but increased with cumulative rainfall, where foliar N content decreased faster for bunch than for lawn grasses (0.36 % at 500 mm) (Fig. 3a).

### Herbivore consumption

**Fig. 4:**
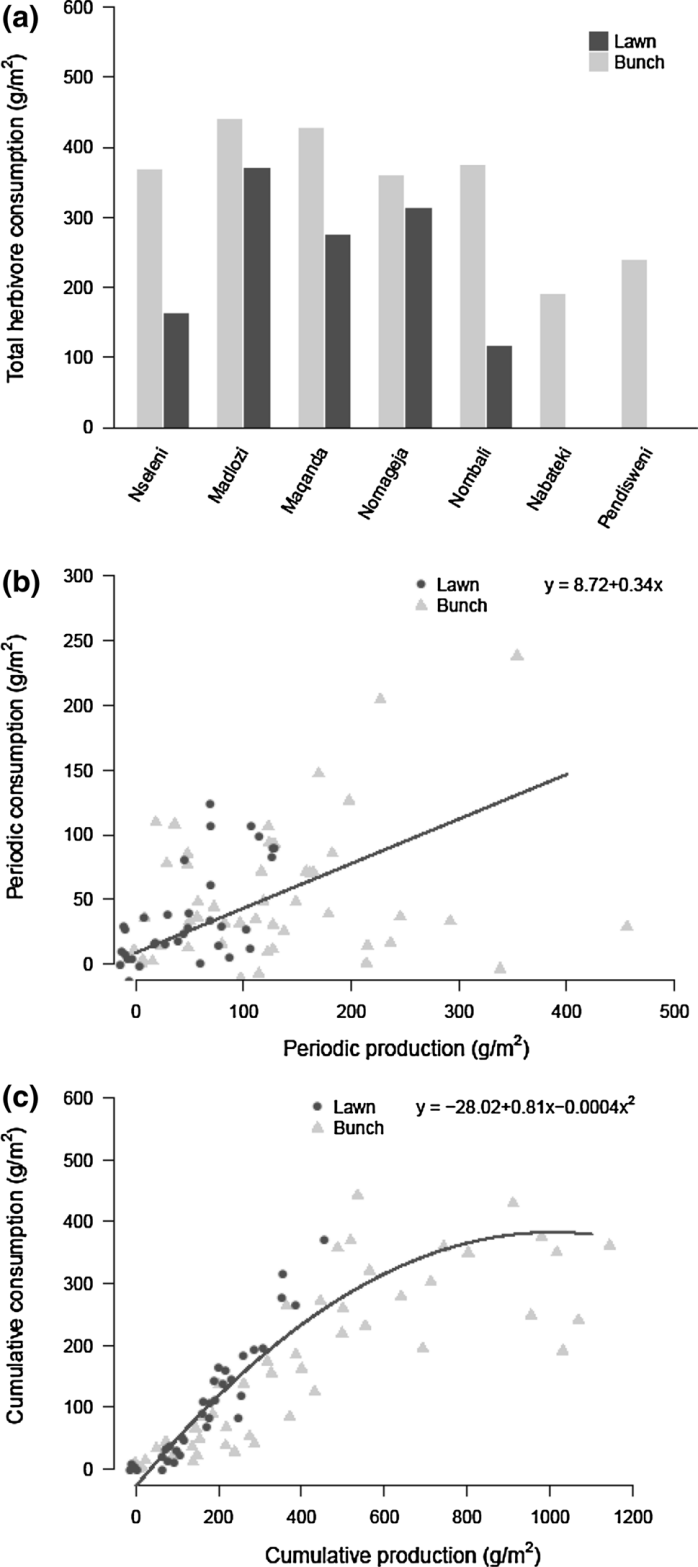
Herbivore consumption for lawn (*black*) and bunch grasses (*grey*) over a full growing season from September 2013 till May 2014. Herbivore consumption was measured using movable cages that were moved every 4–6 weeks. **a** Total herbivore consumption over the growing seasons for each of the seven sites. Sites are ordered by rainfall. **b** Periodic consumption as a function of periodic production. **c** Cumulative consumption against cumulative production. *Solid lines* in **b** and **c** represent both grass vegetation types, as they did not significantly differ from each other

Annually, herbivores consumed more bunch than lawn grasses (Table 1; Fig. 4a). Nevertheless, periodic consumption did not differ between the vegetation types, although it was nearly significant (Fig. 4b, *P* = 0.056). Separate models for lawn and bunch grasses showed a very strong relationship between annual lawn grass production and consumption, but not for bunch grasses (Table 2), which corresponds with the significant interaction term between vegetation type and annual production in the model explaining annual consumption (Table 1). An explanation for this discrepancy between short- and long-term production on consumption rates of bunch grasses can be found in the relationship between cumulative production and consumption (Fig. 4c). There is a strong positive relationship with consumption up to about 500 g m^−2^ grass production, but above that threshold this dependency disappears (Fig. 4c). This indicates a strong relationship between primary production and consumption early in the growing season (low amounts of cumulative rainfall), while later on in the season this relationship is no longer apparent (Table 1). Remarkably, N content negatively affected the consumption by herbivores (Table 1), and this negative effect increased with periodic production (significant interaction).

### Percentage production consumed

**Fig. 5:**
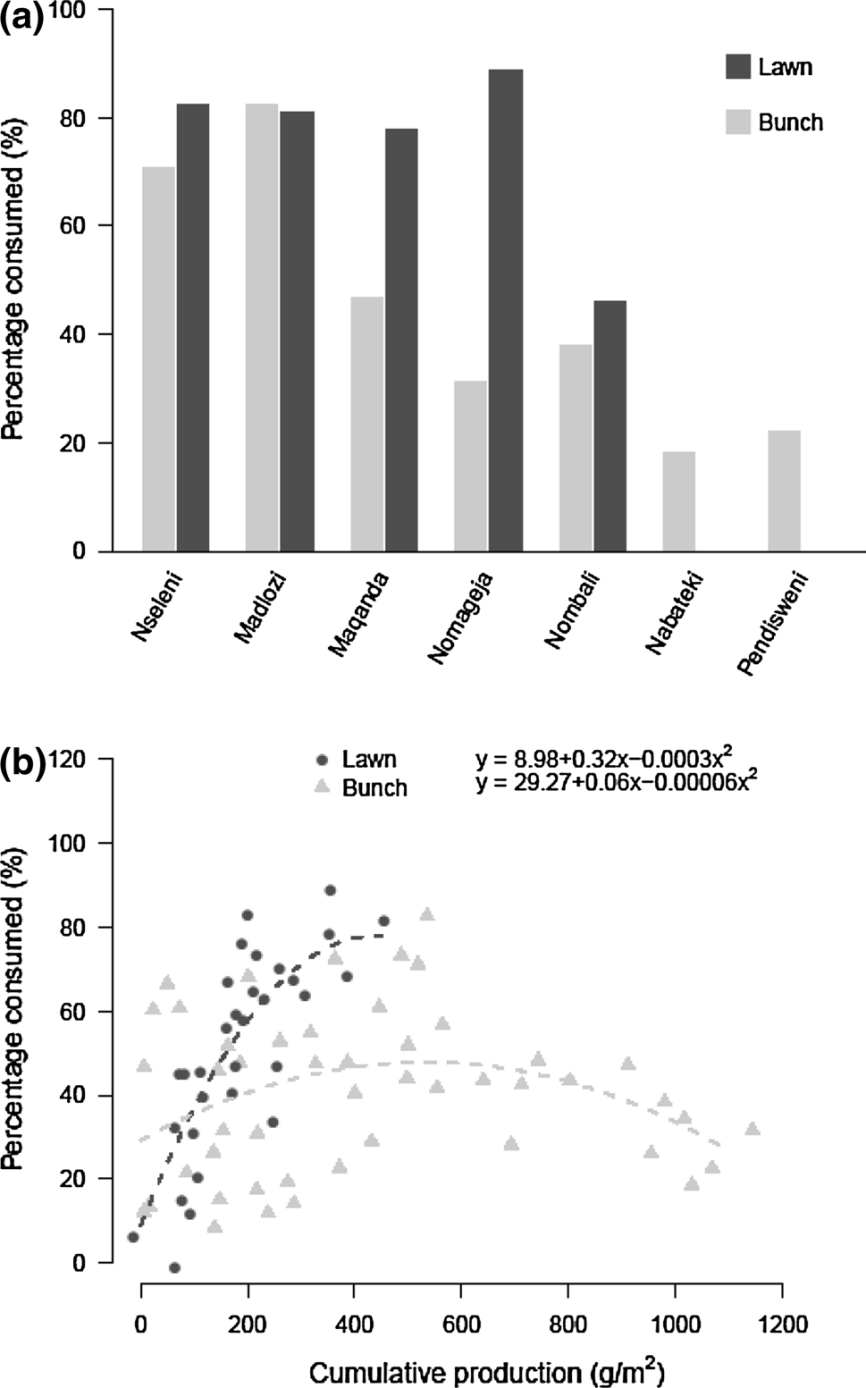
Percentage of the net primary production consumed by large herbivores for lawn (*black*) and bunch grasses (*grey*) over a full growing season from September 2013 till May 2014. **a** Percentage consumed over the full growing season for each of the seven sites. Sites are ordered by rainfall. **b** Percentage primary production consumed by large herbivores as a function of cumulative primary production

The percentage of production consumed by herbivores was higher for lawn grasses than bunch grasses on an annual basis (Fig. 5a; Table 1). On average 75 % of the lawn grass primary production was consumed, compared to 44 % for bunch grasses. Furthermore, primary production negatively affected the percentage consumed on an annual basis (Table 1). Further investigations into the relationship between cumulative primary production and the percentage consumed by herbivores showed a significant interaction between vegetation type and cumulative production (Table 1). Overall, for both vegetation types percentage consumed first increased with cumulative production, but around 500 g m^−2^ this percentage decreased resulting in hump-shaped patterns (Fig. 5b) and significant quadratic term (Table 1). This initial increase of the percentage consumed was stronger for lawn than bunch grasses and did not decrease whereas lawn grasses did not produce more than 500 g m^−2^ during our study, but instead leveled off at ca. 80 % (Fig. 5b).

## Discussion

Our objective was to explore the relative importance of productivity and (quality-driven) consumption differences in determining structural heterogeneity of lawn and bunch grasses in this African savanna. We found that difference in productivity was the main driver of vegetation heterogeneity, where bunch grasses were more productive. Smaller differences were found between the two grass vegetation types in the actual amount of grass consumed, but consumption was higher for bunch grasses, and can, therefore, not explain the spatial heterogeneity in vegetation types. Nevertheless, the percentage of primary production consumed by large herbivores was much higher for lawn grasses, exemplifying their high attractiveness. Similar to findings of earlier studies (McNaughton 1985; O’Connor et al. 2001; Bonnet et al. 2010) we found that periodic primary productivity was strongly dependent on rainfall for both vegetation structural types. In addition, we found a negative effect of cumulative rainfall on grass nutritional quality. Furthermore, consumption by large herbivores seemed mostly limited by primary productivity, but above a threshold of approximately 500 g m^−2^ (only exceeded by bunch grasses, Fig. 4c) consumption rates levelled off.

Our estimates of grazing lawn productivity (0.82 g m^−2^ mm^−1^ rainfall based on periodic rainfall and 0.67 g m^−2^ mm^−1^ rainfall based on cumulative rainfall) were close to those found by Bonnet et al. (2010) (0.77 g m^−^ mm^−1^ rainfall (0.11 × 7 to convert daily to weekly estimates)) but our bunch grasslands were much more productive than lawn grasslands, under similar rainfall conditions. This difference is unlikely to be explained by intrinsic differences between grass functional types, whereas greenhouse studies have shown that under controlled conditions lawn grasses have actually higher relative growth rates (Van der Plas et al. 2013) while showing no differences to bunch grasses in defoliation tolerance (Anderson et al. 2013). Herbivore-induced changes in infiltration and evaporation rates, creating local dry conditions in grazing lawn soils (Veldhuis et al. 2014), may explain their decrease in primary productivity compared to adjacent bunch grass areas. Furthermore, the productivity rates of bunch grasslands that we measured are relatively high compared to other studies (e.g. McNaughton 1985; O’Connor et al. 2001; Knapp et al. 2012). This may be explained by differences in methodology, whereas O’Connor et al. used ungrazed areas to measure productivity and McNaughton used canopy spectroreflectance to estimate changes in above-ground biomass (i.e. productivity). Our moveable exclosure method may be more precise and reflect true productivity values (McNaughton et al. 1996). Furthermore, our study removed all above-ground biomass by means of burning at the start of our study, which may have increased light availability to new growing points and, therefore, increased bunch grass productivity (Knapp and Seastedt 1986; Everson et al. 1988). Finally, reduced consumption of bunch grasses in years without burns might have improved their starting conditions (e.g. nutrients stored in their roots) in our study compared to lawn grasses, whereas grazing during the growing season may strongly reduce grassland productivity in the next growing season (Turner et al. 1993; Ash and McIvor 1998; Knapp et al. 1999; Kirkman 2002).

Differences in consumption rates of different vegetation types are generally explained from plant nutritional value differences. As also found in other studies, we found higher nutritional quality for lawn grasses than for bunch grasses (Stock et al. 2010; Hempson et al. 2014). Furthermore, we found that plant nutritional quality became lower towards higher rainfall, as generally observed in African rangelands and savannas (Breman and Dewit 1983; McNaughton and Banyikwa 1995; Murray 1995; Olff et al. 2002; Coetsee et al. 2011) and declined throughout the growing season. The long-term negative effect of rainfall on nutritional quality can be explained through larger investment in structural plant properties under increased rainfall conditions and plants maturation throughout the season (Olff et al. 2002; Zhang et al. 2013), which is more apparent for bunch than lawn grasses. In addition, the lower decrease in tissue N in lawn grasses with the onset of the dry season may be explained by less nutrient translocation from the leaves in these species.

Large herbivores clearly targeted the biomass produced on grazing lawns as they consumed as became clear from the high percentage biomass consumed, which is as expected due to their higher nutritional quality. This close synchronization between high quality resource production and utilization of grazing lawns indicates their importance to large herbivores (Bonnet et al. 2010). Nevertheless, the actual amount of biomass consumed was higher for bunch grasses. This suggests that it is the low productivity of grazing lawns compared to bunch grasslands determines the difference in vegetation structure, rather than differences in consumption. This does not mean that large herbivores are not important in the formation and maintenance of grazing lawns, which has been repeatedly shown (McNaughton 1984; Cromsigt and Olff 2008).

We chose to study the determinants of spatial heterogeneity in the grass layer of savannas using a burned starting condition. First, this excluded potential differences between sites that were caused by a difference in fire history (and associated nutritional quality). In addition, this represents the situation with the smallest differences in both vegetation height and nutritional quality between lawn and bunch grasses. This allowed us to follow the differentiation in both biomass production and nutritional value and the factors that affect both which was the objective of this study. Nevertheless, starting with an unburned bunch grass layer will likely affect its nutritional value, productivity and consumption. Burned vegetation has higher foliar nutrient concentrations as a result of increased leaf:stem ratios, rejuvenation of plant material and distribution of similar amount of nutrients over less above-ground biomass (Van de Vijver et al. 1999). Therefore, starting with unburned bunch grasses would probably have increased the differences in nutritional quality, which is in line with the patterns we found in this study. Consequently, herbivores are expected to be less attracted to bunch grasses due its lower nutritional value (lower N concentrations) (McNaughton 1985; Moe et al. 1990; Wilsey 1996) and lower mass gains (increased vegetation height) (Anderson et al. 1970; Woolfolk et al. 1975). These effects of fire on grass nutritional quality are generally short-lived (2–3 months) (Van de Vijver et al. 1999) and, therefore, it is expected that consumption rates of unburned bunch grasses might resemble the situation in last months of our study. Effects of fire on grass productivity are mixed, with generally increased productivity in mesic areas (Mott and Andrew 1985; Seastedt et al. 1991; Morgan and Lunt 1999), possibly a result of increased light availability. In contrast, decreased productivity is found in (semi-)arid areas (Scanlan 1980; Hodgkinson 1986; Defosse 1996; Bennett et al. 2003), attributed to increased water stress. Consequently, it is expected that bunch grass primary productivity at our two semi-arid sites would have been higher if we started with unburned bunch grasses and lower at the remaining mesic sites. As the differences that we found between lawn and bunch grasses were smallest at the semi-arid sites and very large at the mesic sites, we don’t expect it would have altered our conclusions, yet remains to be tested.

## Conclusion

Our study highlighted important differences between grazing lawns and bunch grasslands, where bunch grasslands showed much higher productivity, but lower nutritional value. These differences in productivity between lawn and bunch grass-dominated vegetation patches were identified as a more important determinant of this small-scale structural heterogeneity than differences in consumption rates between patch types. Also, both productivity and nutritional quality were strongly affected by rainfall, contributing to spatial and temporal differences in resource heterogeneity.

## Electronic supplementary material

Below is the link to the electronic supplementary material.

Supplementary material 1 (DOCX 767 kb)
