# Supplementary material for: Grassland structural heterogeneity in a savanna is driven more by productivity differences than by consumption differences between lawn and bunch grasses
Source: Oecologia. 2016 Aug 13;182(3):841–53. doi: 10.1007/s00442-016-3698-y (PMC5042998; doi:10.1007/s00442-016-3698-y)
Supplement: Supplementary file 1 — Supplementary material 1 (DOCX 767 kb) [file 442_2016_3698_MOESM1_ESM.docx]

Online Resource 1. Park map showing annual rainfall (left) and the study sites (right).


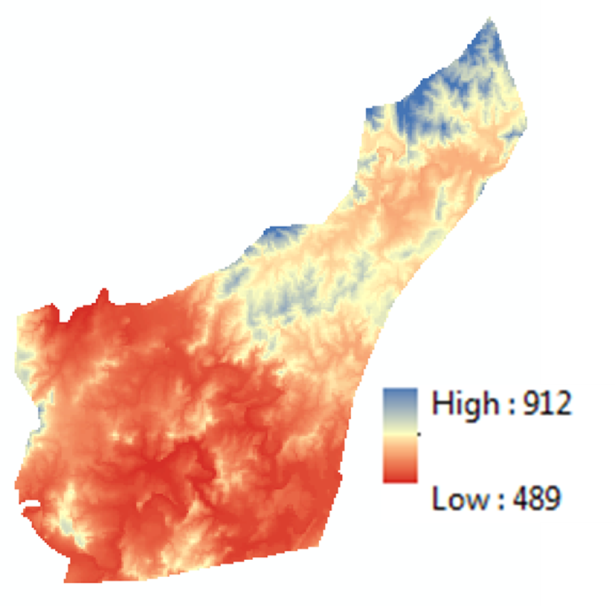

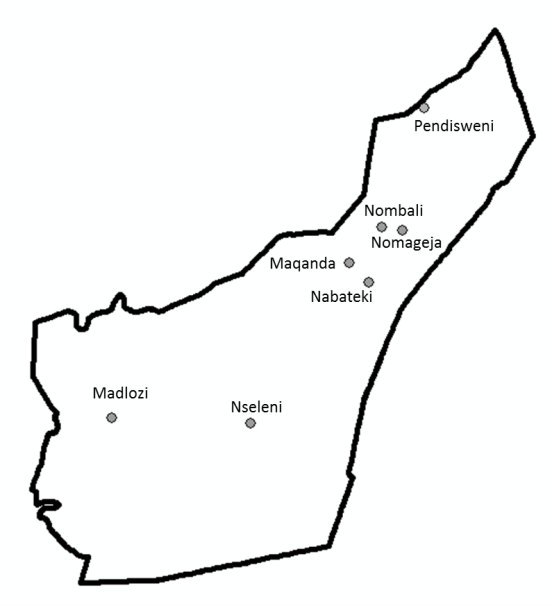


Online Resource 2. Seasonal variation in rainfall, aboveground primary production, nutritional quality and herbivore consumption for lawn and bunch grasses. Different lines indicate averages per site per period where colors represent annual amount of rainfall.
